# Age-adjusted nonparametric detection of differential DNA methylation with case–control designs

**DOI:** 10.1186/1471-2105-14-86

**Published:** 2013-03-06

**Authors:** Hanwen Huang, Zhongxue Chen, Xudong Huang

**Affiliations:** 1 Department of Epidemiology and Biostatistics, University of Georgia, Athens, GA 30605, USA; 2 Department of Epidemiology and Biostatistics, School of Public Health, Indiana University Bloomington, 1025 E. 7th Street, Bloomington, IN 47405, USA; 3 Neurochemistry Laboratory, Department of Psychiatry, Massachusetts General Hospital and Harvard Medical School, Charlestown, MA 02129, USA

**Keywords:** Nonparametric method, One-sided test, Combining p-value

## Abstract

**Background:**

DNA methylation profiles differ among disease types and, therefore, can be used in disease diagnosis. In addition, large-scale whole genome DNA methylation data offer tremendous potential in understanding the role of DNA methylation in normal development and function. However, due to the unique feature of the methylation data, powerful and robust statistical methods are very limited in this area.

**Results:**

In this paper, we proposed and examined a new statistical method to detect differentially methylated loci for case control designs that is fully nonparametric and does not depend on any assumption for the underlying distribution of the data. Moreover, the proposed method adjusts for the age effect that has been shown to be highly correlated with DNA methylation profiles. Using simulation studies and a real data application, we have demonstrated the advantages of our method over existing commonly used methods.

**Conclusions:**

Compared to existing methods, our method improved the detection power for differentially methylated loci for case control designs and controlled the type I error well. Its applications are not limited to methylation data; it can be extended to many other case–control studies.

## Background

Essential for normal development and an influence on a variety of processes related to DNA integrity and function, DNA methylation plays an important role for epigenetic gene regulation in both development and disease [1]. It is associated with processes including genomic imprinting, X-chromosome inactivation, suppression of repetitive elements, and carcinogenesis [2-4]. Aberrant DNA methylation, such as hypomethylation of oncogenes and hypermethylation of tumor suppressor genes, leads to genomic instability and tumorigenesis [5-10].

With the development of high-throughput platforms, genome-wide analysis of large-scale DNA methylation patterns and their impacts on gene regulation have received a significant amount of attention. We are proposing an age-adjusted nonparametric method to detect differentially methylated loci that can account for age effects that has advantages over existing methods the limitations of which we explain next. Typically, methylation levels in Illumina methylation assays are quantified in terms of the *β*-value calculated from the intensity ratio of methylated (M) to unmethylated (U) alleles. Specifically,

$$\beta =\frac{\text{max}\left(M,0\right)}{\text{max}\left(M,0\right)+\text{max}\left(U,0\right)+100},$$

where M and U are the intensities of red and green dyes, respectively, for the GoldenGate and VeraCode Methylation assays, or the signals A and B, respectively, for the Illumina assay. The striking feature of the *β*-values is that they are continuous and range from 0 (totally unmethylated) to 1 (fully methylated).

With more and more DNA methylation data generated from the high-throughput DNA methylation platforms, powerful and efficient statistical methods to handle these complex data are becoming highly demanded. One of the important research topics in this area is to detect differentially methylated loci in case and control studies. The commonly used methods for this purpose are the Student’s *t*-test and linear regression. Recently, a number of model-based approaches have been proposed in the literature. Siegmund [11] introduced a Bernoulli-lognormal mixture model to perform clustering analysis on methylation data generated using MethyLight. Houseman [12] proposed a β-mixture model to classify different tissue types using a recursive-partitioning algorithm for high-dimensional data from Illumina arrays. Wang [13] developed a likelihood based uniform-normal-mixture model to select differentially methylated loci between case and control groups using the Illumina array. The basic idea of Wang [13] is to describe the data using a three-component mixture model and treat completed methylated, unmethylated, and partially methylated loci differently. Wang [13] showed that, compared to the Student’s *t*-test under some situations, their method increases detection power [13]. However, the aforementioned methods assume that the methylation profiles follow some special distributions that are known in terms of a finite number of parameters. Obviously if the underlying true distribution is far from the proposed ones, such assumptions will lead to biased results in practice.

Another complexity of the methylation study comes from the presence of other potential confounders such as age effects. As shown in [14-17], age is by far the strongest demographic risk factor for cancer, and there is substantial evidence that aging affects DNA methylation of specific loci, including cancer-related genes. Based on these observations, it is necessary to adjust age effects in the analysis of detecting differentially methylated loci. If the relationship between the methylation and age is more complex than a linear one, a traditional linear regression leads to inaccurate results. To solve this problem, Chen [16] proposed a method by first dividing the samples into several age groups and then combined the p-values obtained from each individual group together to form a new test. This method has been shown to increase the power in contrast to the traditional *t*-test without age adjustment or regression model with age adjusted linearly. However, within each group, a p-value is obtained from a simple *t*-test that is less robust and thus leaves room for improvement.

In this paper, we propose and examine a novel means to detecting differentially methylated loci and, that is, an age-adjusted nonparametric method that can account for age effects, given that substantial evidence exists that aging affects DNA methylation of specific loci, including cancer-related genes. Our method stems from the rank-based nonparametric methods that focus on a comparison of two entire empirical distribution functions rather than only two means. More specifically, we first divide the subjects into several age groups; then for each group, a nonparametric test is performed on each locus, and an individual p-value is reported. An overall p-value for each locus is estimated through combining all the individual p-values computed previously for that locus in different age groups. Our method does not depend on any distribution assumption but rather adjusts for age effects in an efficient way. We demonstrate the powerfulness of our method using both simulated and real datasets.

## Methods

Assume all the subjects are from *K* different age groups. In the *k*^th^ group, *k = 1,…,K*, there are *n*_*1k*_ control subjects and *n*_*2k*_ case subjects. For each DNA methylation marker, let *y*_*ijk*,_*i = 1,…,n*_*jk*_*, j = 1,2, k = 1,…,K*, denote the observation of β-value for the *i*^th^ subject in *j*^th^ treatment (1 for control and 2 for case) and *k*^th^ age group. To adjust the age influence on the methylation level, the linear model takes the form

$$y_{\text{ijk}}=a_{j}*\text{treatmen}t_{j}+b_{k}*\mathrm{ag}e_{k}+\varepsilon_{\text{ijk}},\text{fori}=1,\ldots ,n_{\text{jk}},j=1,2,k=1,\ldots ,K,$$

Where *a*_*j*_ and *b*_*k*_ are regression coefficients and ε_ijk_ follows a i.i.d normal distribution. The normality assumption is clearly invalid for the β-values which have limited range between 0 and 1. Moreover, the relationship between the β-value and age is likely to be more complicated than a linear one. Therefore more powerful and robust nonparametric methods are desirable.

Here we propose a new nonparametric method which does not depend on any distribution assumption and meanwhile allows for the adjustment of covariates. We process as follows. For each age group *k (k = 1,…,K)*, we test the difference between the control group *y*_*i1k*_*(i = 1,…,n*_*1k*_*)* and the case group *y*_*i2k*_*(i = 1,…,n*_*2k*_*)*. Our goal is to test whether or not the two methylation groups follow the same distribution. Toward this end, the Wilcoxon rank sum test is a useful tool when there are reasons to believe that the outcome variables of interest may fail certain distributional assumptions required for parametric methods. However, as discussed in Baumgartner [18], Wilcoxon rank sum test is not suitable for situations where the expected values of the two populations are close to each other. To overcome this problem, they proposed a more powerful nonparametric test to handle the general two-sided two-sample problem [18]. Neuhaeuser further extended the two-sided two-sample test to a one-sided test that can detect if one population is stochastically larger than the other [19]. Our p-value calculation for each age group is based on Neuhaeuser’s one-sided test whose statistics can be explicitly formulated as

$$B_{k}=\frac{1}{2}\left(B_{1k}-B_{2k}\right),$$

where

$$B_{1k}=\frac{1}{n_{1k}}\overset{n_{1k}}{\underset{i=1}{\sum }}\;\frac{\left(G_{i}-\frac{n_{1k}+n_{2k}}{n_{1k}}i\right)|G_{i}-\frac{n_{1k}+n_{2k}}{n_{1k}}i|}{\frac{i}{n_{1k}+1}\left(1-\frac{i}{n_{1k}+1}\right)\frac{n_{2k}\left(n_{1k}+n_{2k}\right)}{n_{1k}}}$$

$$B_{2k}=\frac{1}{n_{2k}}\overset{n_{2k}}{\underset{j=1}{\sum }}\;\frac{\left(H_{j}-\frac{n_{1k}+n_{2k}}{n_{2k}}j\right)|H_{j}-\frac{n_{1k}+n_{2k}}{n_{2k}}j|}{\frac{j}{n_{2k}+1}\left(1-\frac{j}{n_{2k}+1}\right)\frac{n_{1k}\left(n_{1k}+n_{2k}\right)}{n_{2k}}}$$

Here *G*_*i*_, *i = 1,2,…, n*_*1k*_ and *H*_*j*_*,j* = 1*,*2*,*…*,n*_*2k*_ are the ranks of the samples from the *k*^th^ case and control groups, respectively. Due to the intractable asymptotic distribution for the test statistics *B*, it is hard to find a close form for the relationship between p-value and *B*. We will use numerical fit to approximate this relationship. We first obtain the empirical distribution of *B* based on 10^7^ permutations and then fit the distribution function piecewise exponentially to obtain the empirical relationship. The p-value for the *k*^th^ age group can be calculated according to this empirical formula.

As a consequence, we have *K* p-values from the left-sided test, denoted by *p*_*lk*_*(k = 1,…,K)*, and *K* p-values from the right-sided test, denoted by *p*_*rk*_ *= 1-p*_*lk*_*(k = 1,…,K).* Then combining *K* left-sided p-values together gives a statistic

$$T_{l}=-2\overset{K}{\underset{k=1}{\sum \;}}\log\left(p_{\mathrm{lk}}\right)$$

Similarly, combining *K* right-sided p-values together gives a statistic

$$T_{r}=-2\overset{K}{\underset{k=1}{\sum \;}}\text{log}\left(p_{\text{rk}}\right)$$

Under the null hypothesis of no difference between the two treatment groups, *p*_*lk*_ and *p*_*rk*_ are uniform [0,1] random variables for *k = 1,…,K*. Therefore, according to Fisher [20], both *T*_*l*_ and *T*_*r*_ will follow a chi-square distribution with degree of freedom *2 K*. We define a new variable:

$$T=\text{max}\left\{T_{l},T_{r}\right\},$$

then we have [21]

$$2\alpha -\alpha^{2}\leq \mathrm{Pr}\left(T>x\right)\leq 2\alpha ,\;\text{where}\;\alpha =1-F_{X_{2K}^{2}}\left(x\right),\;\text{and}\;F_{\chi_{2K}^{2}}\;\text{is}\;\text{the}\;\text{CDF}\;\text{of}\;\chi_{2K}^{2}.$$

Thus, for small α, we can approximate the p-value of *T* by its upper bound 2α as

$$\mathrm{Pr}\left(T>x\right)\approx 2\alpha$$

More details can be found in [21-23]. For large α, we will fit a formula empirically through permutation. We call the above proposed method “combined Baumgartner-Weiß-Schindler (BWS) test”.

## Results and discussion

### Empirical fit of the p-value formula for one-sided BWS test

The asymptotic distribution function of *B* is complex and in practice permutation method is often used. The permutation results depend on the sample size. But as mentioned in Baumgartner [18], for a two-sided test, the asymptotic distribution can be approximated by the permutation method quite well even with a small sample size (as small as 10). We first derive the empirical formula to fit the asymptotic distribution using the permutation method. Toward this end, we sample two subpopulations from the same distribution (e.g. standard normal), each of which has a sample size of 30. Then, the whole populations are permuted 10^7^ times, and a one-sided BWS test statistic *B* is calculated for each permuted sample. Then we fit the empirical cumulative distribution of *B* using a piecewise exponential function to arrive at the following empirical formula

$$\begin{array}{l} P\left(B\right)=\{\begin{array}{c} e^{-0.699-1.255*B}\;\left(0\leq B<1.5\right)\\ e^{-0.895-1.153*B+0.0173*B^{2}}\left(1.5\leq B<9\right),\\ e^{-2.895-0.786*B}\left(B\geq 9\right) \end{array}\;\mathrm{and}\\ P\left(-B\right)=1-P\left(B\right). \end{array}$$

The node points we used here are 1.5 and 9. We find that the choice of node points has very little influence on the final analysis results for both simulated and real data. Note that the sample size we used for deriving this formula is 30. We have also tried some other choices and found that the results are quite similar. Thus, we recommend the above formula to be used in practice for problems with a sample size larger than 10.

### Simulation results

The first simulation settings are for the evaluation of the type I error rate for the proposed method. For the purposes of comparison, we also include the results from the combined *t*-test proposed in [16], linear regression and combined Wilcoxon methods for all simulated and real datasets. We assume that there are 6 age groups, and each group includes 100 subjects, 50 controls and 50 cases. For each age group, we also assume the β-values follow a three-component mixture distribution as in Wang [13]. Let τ_1_ and τ_2_ be the two threshold values. The first and the second components are uniform distributions

$$U_{\left[0,\tau_{1}\right]}$$

and

$$U_{\left[\tau_{2,}1\right]}$$.

The third component is a truncated normal distribution

$$N_{\left[\tau_{1},\tau_{2}\right]}\left(\mu ,\sigma^{2}\right)$$.

The probabilities for a measurement to fall into these three components are *π*_*1*_*, π*_*2*_*, π*_*3*_ respectively. Under the null hypothesis, the two treatment groups are sampled from the same distribution. The mean of the truncated normal distribution is taken to be μ + k*δ_*μ*_ for the *k*^th^ age group. The simulation is repeated 10000 times. Table 1 lists the proportion of times that null is rejected using the four different methods under different parameter settings. Table 1 shows that the nominal type I error rate of 0.05 is well controlled by all methods.

**Table 1:** **Estimated Type I error rates at significant level 0.05 based on the four methods under different parameter settings of the three-component mixture distributions with *τ***_***1***_ **= 0.1, *τ***_***2***_ **= 0.9 and *δ***_***μ***_ **= 0.05**

**Parameter Setting**	***π***_***1***_	***π***_***2***_	***π***_***3***_	***μ***	***σ***	***t*-test**	**Linear regression**	**Wilcox**	**BWS**
1	0.3	0.5	0.2	0.3	0.1	0.0513	0.0521	0.0514	0.0458
2	0.4	0.5	0.1	0.2	0.1	0.0495	0.0494	0.0519	0.0492
3	0.4	0.5	0.1	0.3	0.2	0.0511	0.0519	0.0503	0.0495
4	0.5	0.1	0.4	0.3	0.2	0.0528	0.0521	0.0544	0.0511
5	0.4	0.2	0.4	0.2	0.1	0.0509	0.0510	0.0472	0.0464

The second simulation settings are for assessing the power of the proposed method under alternative hypothesis, i.e. the case and control subjects are sampled from different distributions. We still assume that the β-values follow the similar three-component mixture distributions. Two scenarios are considered here. In the first scenario, we use different means for different treatment groups. More specifically, we let the truncated normal mean for the control sample be constant *μ*, but for the case sample vary as *µ* + *k* * *δ*_*μ*_ for the *k*^th^ age group. We replicate the simulation 10000 times for each scenario. The power is defined as the proportion of times that the p-value is less than 0.05. The first two rows in Table 2 list the results for this scenario. In the second scenario, we let the two threshold values vary as *τ*_*1*_ *+ kδ*_*τ*_ and *τ*_*2*_*-kδ*_*τ*_ for the *k*^th^ case group but keep them constants as *τ*_*1*_ and *τ*_*2*_ for all control groups. The results are listed in the last two rows of Table 2. For the first scenario, the mean values are different for the case and control groups. As expected, all four methods have increasing powers as the signal increases. For the second scenario, the expected values are the same, but the variances are different between the two treatment groups. In this situation, our proposed method is more powerful in detecting the difference than the other three methods. Therefore, the proposed method can detect not only the location difference but also the scale difference between the two distributions.

**Table 2:** **Estimated powers of the four methods at significant level 0.05 under different parameter settings for the three-component mixture distributions with *τ***_***1***_ **= 0.1, *τ***_***2***_ **= 0.9**

**Parameter Setting**	***π***_***1***_	***π***_***2***_	***π***_***3***_	***μ***	***σ***	***t*-test**	**Linear regression**	**Wilcox**	**BWS**
*δ*_*μ*_ = 0.03	0.3	0.5	0.2	0.3	0.1	0.475	0.479	0.749	0.836
*δ*_*μ*_ = 0.05	0.3	0.5	0.2	0.3	0.1	0.889	0.892	0.951	0.988
*δ*_*τ*_ = 0.03	0.45	0.1	0.45	0.5	0.05	0.048	0.047	0.078	0.727
*δ*_*τ*_ = 0.06	0.45	0.1	0.45	0.5	0.05	0.048	0.047	0.092	0.877

The third settings assume that the β-values for both the case and control subjects follow the beta-distributions. Let s_1_ = s_2_ = 4. For the case group, the β-values are sampled from a beta-distribution *Beta*(*s*_1_ + *δ*, *s*_2_ − *δ*). For the control group, the β-values are sampled from a beta-distribution *Beta*(5*s*_1_ + *δ*, 5*s*_2_ − *δ*). Here *δ* takes six different values, *-3λ, -2λ, -λ, 0.5λ, λ,* and *1.5λ*, one for each age group.

Based on the above setting, it can be easily shown that the mean of the distribution for the case group is

$$\frac{s_{1}+\delta }{s_{1}+s_{2}}$$

while the mean of the distribution for the control group is

$$\frac{s_{1}+\delta /5}{s_{1}+s_{2}}$$

The mean difference between the two treatment groups is

$$\frac{4\delta /5}{s_{1}+s_{2}}$$

which will increase with *δ*. Table 3 lists the empirical powers of four methods for different *λ* values. In all situations, the most powerful method is combined DWS. Since the variance of two distributions are different, even in situation where *δ = 0*, the power of the proposed method can still reach 0.67, whereas the other three methods have no power at all. As *λ* decreases, the mean differences become smaller and make it more difficult to distinguish between the two treatment groups. As expected, the powers decrease for all methods as *λ* decreases. For small *λ*, the power from the combined DWS method is much bigger than those from the other three methods. The performances of the combined *t*-test and combined Wilcoxon methods are quite similar, and both are much better than the linear regression method.

**Table 3:** **Change of the power with *λ *for four different methods when the distributions are *Beta*(*s***_**1**_ **+** ***δ*, *s***_**2**_ **−** ***δ*) and *Beta*(5** ***s***_**1**_ **−** ***δ*, 5** ***s***_**2**_ **+** ***δ*) for the case and control groups; *δ *takes the values of *-3λ, -2λ, -λ, 0.5λ, λ*, and *1.5λ *for the six age groups and s**_**1**_ **= s**_**2**_ **= 4**

*** λ***	**Combined *t*-test**	**Linear regression**	**Combined Wilcoxon**	**Combined BWS**
0	0.056	0.053	0.073	0.669
0.05	0.075	0.057	0.094	0.703
0.1	0.182	0.087	0.200	0.832
0.15	0.402	0.141	0.412	0.939
0.2	0.701	0.212	0.687	0.988
0.25	0.911	0.298	0.893	0.999

### Real data results

We also applied our proposed method to the United Kingdom Ovarian Cancer Population Study (UKOPS) [15] to select differentially methylated loci between ovarian cancer cases and healthy controls. The data were created using Illumina Infinium Human Methylation27 Beadchip and downloaded from the NCBI Gene Expression Omnibus ( http://www.ncbi.nlm.nih.gov/geo) under the accession number GSE19711. There were 274 control samples and 131 pre-treatment case samples, and the number of loci was 27578. For the data quality control, we removed 29 patients (15 controls and 14 treatments) with BS conversion efficiency value < 4000 or coverage rate < 95% [15]. For each treatment group, the samples were divided into 6 age groups (50–55, 55–60, 60–65, 65–70, 70–75, and 75 and over). We further removed 12 patients in the pre-treatment group whose ages exceeded 78 since there were no such patients in the control group. The final sample size for each individual group is the same as the one listed in Table 2 of Chen [16] except that the “75 and over” group has 13 pre-treatment samples instead of 25. This dataset was analyzed by both Wang [13] and Chen [16] in their papers. Wang [13] did not consider the age effect, and only 96 cases and 136 controls were included in their analysis after further screening; while Chen [16] included the 12 patients with ages exceeding 78; thus their results are different from ours even though the same method was used.

**Figure 1:**
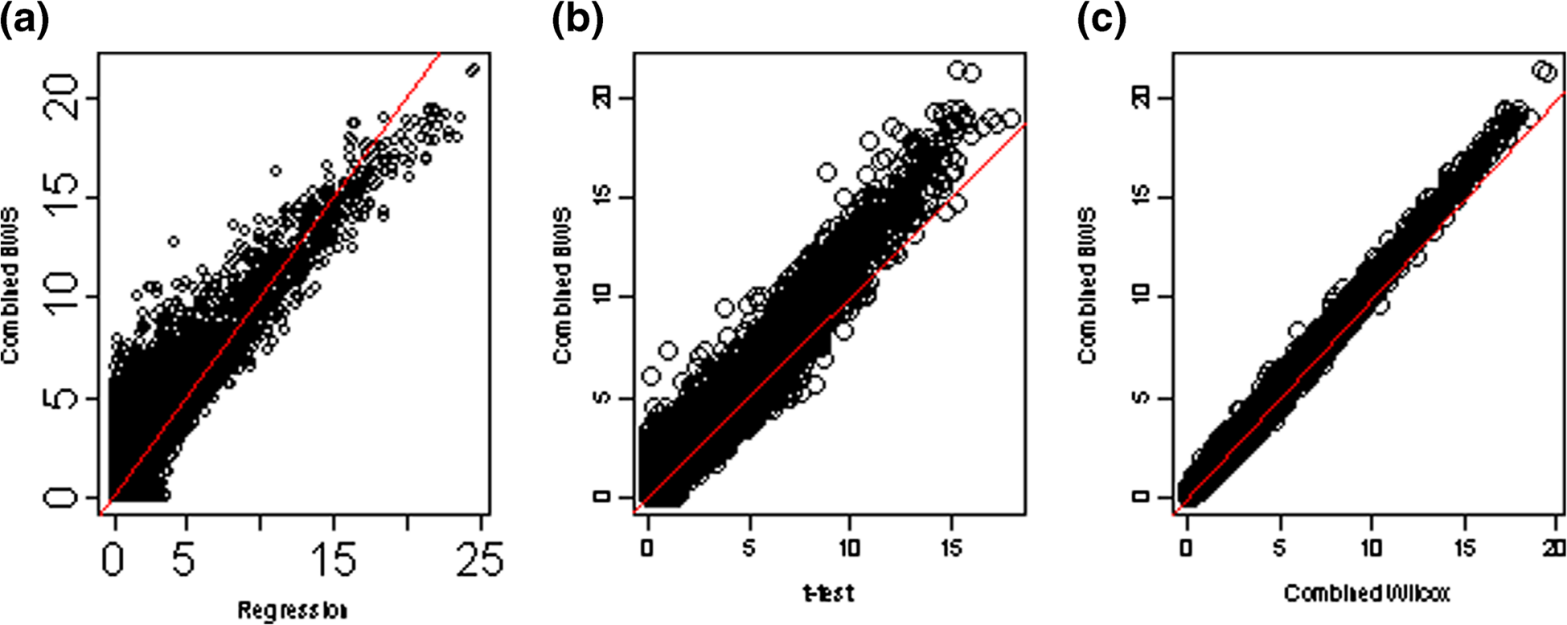
**Scatter plots for negative *log10 *p-values based on different methods.** The left panel is for the comparison between combined DWS and linear regression. The middle panel is for the comparison between combined DWS and combined *t*-test. The right panel is for the comparison between combined DWS and combined Wilcoxon test.

Figure 1 shows the scatter plots for each locus based on the negative *log10* p-values derived from four different methods. Figure 1 (a) plots the results for the combined DWS test and the linear regression method, Figure 1 (b) plots the results for the combined DWS test and combined *t*-test, Figure 1 (c) plots the results for the combined DWS test and combined Wilcoxon-test. From Figure 1 (a), it can be seen that most of the loci with small p-value in the linear regression tend to have small p-value in our proposed method as well. However, there exist many loci whose p-values are large in the linear regression but small and significant in the combined DWS test, i.e., those points in Figure 1 (a) with x-values close to zero but y-values large than 3. This indicated that our proposed method is more powerful than the linear regression method in detecting the differentially methylated loci. Similar conclusions can be drawn from Figure 1 (b) and Figure 1 (c) for the comparison with the combined *t*-test and combined Wilconxon test respectively.

Table 4 lists the number of the loci detected by each of the four tests based on four different significant levels, 10^-3^, 10^-4^, 10^-5^, 10^-6^. Clearly at the same significant level, in terms of the number of significant loci detected, the most powerful method is combined DWS, the next one is combined Wilcoxon test, and then combined *t*-test and linear regression. Table 4 also reports the numbers of significant loci obtained by pairs of the proposed test and each of the other three methods for given significance levels. For example, when the cutoff p-value is 10^-3^, the combined BWS, the linear regression, the combined *t*-test, and the combined Wilcoxon obtained 3387, 2038, 2754, and 3143 significant loci, respectively. However, among those 3387 loci that have p-values less than 10^-3^ using the combined BWS method, there were 1884, 2659, and 3081 loci overlapping with the linear regression, the combined *t*-test, and the combined Wilcoxon methods, respectively. In other words, there were 1503, 728, and 306 significant loci were only obtained by the proposed test, but not by the linear regression, the combined *t*-test, and the combined Wilcoxon, respectively. In contrast, at the same significance level 10^-3^, there were only 154, 95, and 62 loci whose p-values from the new method are larger than 10^-3^ but less than 10^-3^ from the linear regression, the combined *t*-test, and the combined Wilcoxon, respectively. Therefore most of the loci detected by the other three methods are also detected by the proposed method. However, for the same cutoff level, there were many loci that were significant in the proposed method but not in the other three methods, a point that clearly demonstrated the advantages of our method over those three.

**Table 4:** Number of loci with p-values less than the given cutoff significance levels from different methods

**Cutoff p-value**	**Linear regression (I)**	**Combined *t*-test (II)**	**Combined Wilcoxon test (III)**	**Combined BWS test (IV)**	**From both I and IV**	**From both II and IV**	**From both III and IV**
10^-3^	2038	2754	3143	3387	1884	2659	3081
10^-4^	1438	1879	2152	2321	1352	1795	2117
10^-5^	1120	1343	1495	1653	1059	1286	1479
10^-6^	894	982	1109	1222	844	931	1099

### Discussion

**Figure 2:**
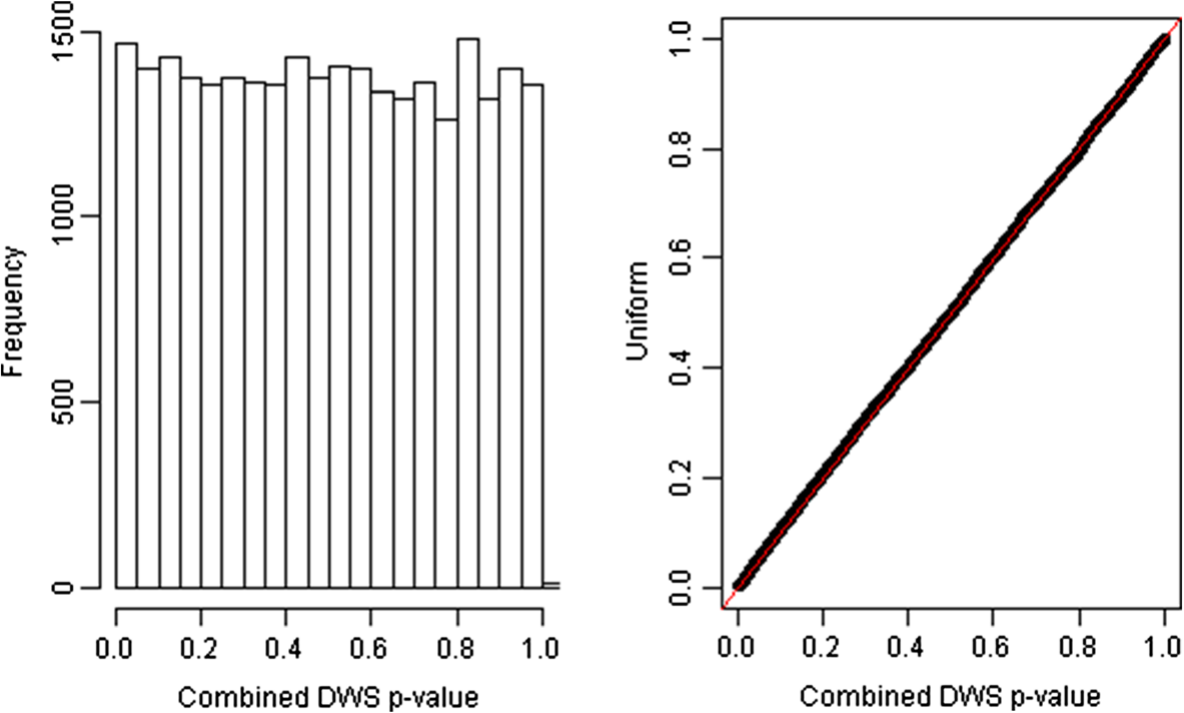
**Test the distribution of p-values by applying the proposed method to a newly created case–control data based on the samples from the original control group.** The left panel is for the histogram and the right is a qq-plot against the uniform [0,1] distribution.

To study whether or not the proposed method can control type I error rate as well, we created pseudo case and control samples. The way we did this was to first randomly divide the original control subjects into two parts for each age group. Then we put one part into the new pseudo-control group and the other one into the new pseudo-case group. The distribution of p-values from applying the proposed method to this new case–control data set is shown in Figure 2 (a). It is very close to uniform distribution, and this finding is further confirmed by the qq-plot against the uniform [0,1] distribution as illustrated in Figure 2(b). Therefore, our method increased the detection power while it simultaneously controlled the type I error rate.

In this paper we chose different cutoff p-values to compare the performance of the proposed test with others. We did not consider the multiple testing issue, which is an important but difficult task for this area [4] and other areas where a large number of correlated variables are tested simultaneously [24-28]. The traditional correction methods for multiple comparisons, such as Bonferroni correction, are inappropriate here as they are too conservative due to the fact that many loci from the same gene are highly positively correlated. To account for the correlations among loci, methods based on the concept of “effective number” may be adopted [29].

There are many ways to combine p-values from independent tests [20,30-32]. In this paper, we chose to use Fisher test due to its robustness. It is possible, however, that other methods may be more powerful under some certain conditions. The combined p-value method used in this paper is based on the assumption that the test for every individual age group is independent from each other. However, if this assumption is questionable, the current combined p-value method needs to be modified such that it can handle the correlations among the individual tests as well. This is another research topic we will pursue in a future paper.

## Conclusions

In case–control methylation studies, the underlying distribution of the β-values is rarely known in advance, and clearly the normality assumption is not valid. Parametric models can be powerful if the assumptions for the models are valid, but they can also lead to biased results if the underlying true distribution is far deviated from the imposed ones. Thus, it is desirable to choose a powerful yet robust statistical test that does not depend on any underlying distribution assumption. In this article we proposed and examined a rank-based nonparametric method to detect differentially methylated loci. Through simulation, we showed that our proposed method is as powerful as the linear regression, *t*-test and Wilcoxon rank sum test methods if the mean differences between the two treatment groups are large. However, our method substantially outperformed the others in situations where the mean differences between the two groups were small while the variance differences were large.

Note that the age effects are strongly associated with methylation, and the ignoring age effect will cause a loss of power or a large number of false positives. Another advantage of the proposed method over many existed ones is that it combined the nonparametric test together with age adjustment. Our next goal was to generalize our method to adjust for more confounders other than the age such that it can have a much wider application in methylation research.

## Competing interests

The authors declare that they have no competing interests.

## Authors’ contributions

HH devised the basic idea, analyzed the data and drafted the manuscript; ZC devised the basic idea and analyzed the data. XH participated in the study design, discussion and edit the manuscript. All authors read and approve the final manuscript.
